# Anabolic androgenic steroids may be associated with early coronary artery disease

**Published:** 2018

Anabolic androgenic steroids may be associated with early coronary artery disease, according to research presented at the Brazilian Congress of Cardiology (SBC 2017). The annual congress of the Brazilian Society of Cardiology (SBC) was held in São Paulo from 3 to 5 November 2017. Experts from the European Society of Cardiology (ESC) presented a special programme.

‘Anabolic androgenic steroid abuse among young people is a widespread problem worldwide, and adverse events such as sudden cardiac death and heart attack have been reported in athletes,’ said lead author Francis Ribeiro de Souza, PhD student, Heart Institute (Instituto do Coracao; InCor), Medical School, University of Sao Paulo (Hospital das Clinicas da Faculdade de Medicina da Universidade de Sao Paulo; HCFMUSP), Brazil. ‘In Brazil, around one million people have used anabolic androgenic steroids at least once, and they are the seventh most commonly used drug in the country,’ he added.

This study examined whether anabolic androgenic steroids could be associated with early coronary artery disease. It also tested whether reduced high–density lipoprotein (HDL) function could be a mechanism leading to coronary artery disease in anabolic androgenic steroid users.

The study included 51 men with an average age of 29 years (range 23–43 years). Of those, 21 did weight lifting and had taken anabolic androgenic steroids for at least two years, 20 did weight lifting but did not take steroids, and 10 were healthy but sedentary.

Participants underwent computed tomography coronary angiography (a type of imaging used to visualise the arteries) to assess the presence of atherosclerosis in the coronary arteries.

A urine test was performed in all participants to confirm steroid use. Blood samples were taken to measure lipid levels including HDL. The researchers used cell cultures to measure the ability of each participant’s HDL to perform its normal function of removing cholesterol from the macrophages.

The researchers found that 24% of steroid users had atherosclerosis in their coronary arteries, compared to none of the non–users and sedentary participants. The steroid users with atherosclerosis also had significantly reduced HDL levels and HDL function.

Mr Ribeiro de Souza said: ‘Our study suggests that anabolic androgenic steroid use may be associated with the development of coronary artery disease in apparently healthy young people. Steroids may have an impact on the ability of HDL to remove cholesterol from macrophages, thereby promoting atherosclerosis.’

‘This was a small, observational study and we cannot conclude that steroid use causes atherosclerosis,’ he continued. ‘Larger studies with longer follow up are needed to confirm these results.’

Mr Ribeiro de Souza concluded: ‘We observed coronary atherosclerosis in young anabolic androgenic steroid users, which in combination with lower HDL levels and reduced HDL function, could increase the risk of cardiovascular events. Greater awareness is needed of the potential risks of these drugs.’

Dr Raul Santos, scientific chair of SBC 2017, said: ‘This study, despite its small sample size, is well done and calls attention to a possible important health problem in Brazil and elsewhere, since it shows not only the classical lipid disturbances induced by steroids but actually associates them with subclinical atherosclerosis presence, something that we are not supposed to find in young individuals.’

Professor Fausto Pinto, ESC immediate past president and course director of the ESC programme in Brazil, said: ‘This is an important issue in cardiovascular prevention, which deserves further study. During SBC 2017, ESC experts highlighted hot topics in prevention and other fields of cardiology that were presented at ESC Congress 2017 in Barcelona.
